# Children's environmental health: an under-recognised area in paediatric health care

**DOI:** 10.1186/1471-2431-9-10

**Published:** 2009-02-06

**Authors:** Tania G Gavidia, Jenny Pronczuk de Garbino, Peter D Sly

**Affiliations:** 1 WHO Collaborating Centre for Research on Children's Environmental Health, Curtin University of Technology, Perth, WA, Australia; 2 Department of Public Health and Environment, World Health Organization, Geneva, Switzerland; 3 Centre for Child Health Research, University of Western Australia, Perth, WA, Australia

## Abstract

The knowledge that the environment in which we live, grow and play, can have negative or positive impacts on our health and development is not new. However the recognition that adverse environments can significantly and specifically affect the growth and development of a child from early intrauterine life through to adolescence, as well as impact their health later in adulthood, is relatively recent and has not fully reached health care providers involved in paediatric care.

Over the past 15 years, world declarations and statements on children's rights, sustainable development, chemical safety and most recently climate change, have succeeded in cultivating a global focus on children's health and their right to a healthy environment. Many international calls for research in the area, have also been able to identify patterns of environmental diseases in children, assess children's exposures to many environmental toxicants, identify developmental periods of vulnerability, and quantify the cost benefits to public health systems and beyond, of addressing environmentally related diseases in children. Transferring this information to front-line health care providers and increasing their awareness about the global burden of disease attributed to the environment and children's especial vulnerability to environmental threats is the salient aim of this commentary.

## Commentary

Children's exposure to environmental toxicants is different from those of adults because of differences in physiology and behaviours. In relation to body weight, children drink more water, eat more food and breathe more air than adults and will therefore have substantially heavier exposures than adults to any toxicants that are present in water, food or air. Their hand to mouth behaviour, and the fact that they live and play close to the ground further increases children's exposure to environmental threats [1]. Time spent in different settings, such as the home, the playground, school and the wider community may also cause illnesses, depending on the children's and adolescents' own predisposition to chemical, physical, and biological risks [2].

Exposure to environmental agents is the first step in the sequence of environmentally related health effects. In recent decades, new knowledge has emerged about the special vulnerability of children to environmental exposures. Yet, despite the efforts of international organizations, such as the World Health Organization (WHO) and the United Nations Environment Programme (UNEP), the environmental burden of paediatric disease is not well recognised globally amongst paediatric health care providers in the front line.

In 2006, a seminal report published by the WHO examined how specific diseases and injuries are impacted by environmental risk, at the global, regional and population level. The report entitled: *Preventing Disease Through Healthy Environments: Towards an estimate of the environmental burden of disease*, estimated that about 23% of all deaths worldwide and 36% of deaths among children 0–14 years of age can be attributed to environmental toxicants [3]. Major inequalities in social, economic, health, and environmental well-being between developed and developing countries means that much of this burden rests upon the poor and vulnerable, with 25% of all deaths in children in developing regions attributable to environmental causes, as opposed to only 17% in developed regions [3].

The report further estimated that children under 5 years of age suffer disproportionately from environmental hazards, with 36% of the overall disease burden and 37% of deaths attributable to modifiable environmental causes. In terms of just diarrhoea and lower respiratory infections, two of the most significant childhood killers, environmental interventions could prevent the deaths of over 2 million children under the age of five every year [3]. Preventing negative environmental exposures therefore, could save more than four million lives a year in children alone, mostly in developing countries [4].

Whether children are affected by *traditional*, *modern *or *emerging *environmental threats, all significantly contribute to the burden of death, disease and disability. While developing nations carry a disproportionate heavy share of the burden, it would be incorrect to assume environmental threats to health in industrialised nations are not a concern [3]. *Traditional risks *such as unsafe drinking water, poor sanitation, indoor air pollution from household solid fuel use, diarrhoeal, infectious and vector borne diseases, poor food hygiene and poor quality housing, continue to account for the majority of deaths and illnesses around the world[5]. In wealthier countries, *modern *environmental threats to health generally stem from industrialization, such as pollution from industry and transport and uncontrolled urbanization[5]. In addition to traditional and modern risks, there also risks that are only emerging and their effects remain speculative. Transboundary contamination by persistent toxic substances (PTS), ozone depletion and hence ultraviolet (UV) and ionising radiation, global climate change, and exposure to chemicals that can disrupt endocrine (endocrine disrupting chemicals – EDC) and autoimmune functions have been identified as *emerging threats *to children residing in both developed and developing countries[6].

It is widely accepted nowadays that children have a special vulnerability to environmental pollution because their body is still developing[7]. In particular the developmental and growth processes in foetus, infant, child, and adolescent life can define periods of great vulnerability to environmental toxicants due to the rapid tissues and organs growth, development and differentiation until maturity. Exposure to a wide range of chemicals and environmental toxicants during this period has the potential to significantly affect the development, maturation, growth and function of organ systems well into adulthood[6,7].

More than three million children under five die each year from environment-related causes and conditions. This makes the environment one of the most critical contributors to the global toll of more than ten million child deaths annually[8]. Despite the alarming toll of the environment on children's health and vast amount of evidence supporting the notion that children's health is adversely affected by the environment in which children live grow and play, children's environmental health remains an under-recognised area in paediatric health care.

## What does the Future Hold?

In 2004, the top five causes of death in low-income countries were pneumonia, followed by heart disease, diarrhoea, HIV/AIDS and stroke. In high-income countries, the list was lead by heart disease, followed by stroke, lung cancer, pneumonia, and asthma/bronchitis[9]. Due to the expected economic growth in low and middle-income countries, globally by 2030, the four leading causes of death are predicted to be ischeamic heart disease, cerebrovascuar diseases (stroke), chronic obstructive pulmonary disease (COPD), and lower respiratory infections (mainly pneumonia)[9]; environmental exposures contribute significantly to these diseases.

Cardiovascular disease for example, such as ischaemic heart and cerebrovascular (stroke) diseases have been extensively associated with air pollution, workplace exposure to chemicals, such as lead, and exposure to environmental tobacco smoke (ETS), resulting in a total of 16% of the total burden of cardiovascular disease attributable to the environment, corresponding to 2.5 million deaths per year which could be prevented [3]. In terms of total global disease burden of COPD, an estimated 42% of the burden can be attributed to the environment[3]. Finally after combining the effects of indoor and outdoor air pollution and other indoor conditions, at least 42% of all lower respiratory infections can also be attributable to the environment in developing countries, while in developed lower respiratory infections countries, this rate is halved to 20%[3]. Measured in disability-adjusted life years (DALY's), 36% of the global disease and injury burden falls on children under the age of 15, however in low-income countries, children are the bearers of more than 50% of the disease burden[9].

The burden attributable to the environment of such conditions, are avoidable and preventable, highlighting the full potential for environmental intervention to improve human health through effective management of the environments in which we live, grow and play, rather than simply treating diseases and ailments after they've occurred.

## What to do about Children's Environmental Health

Health professionals in the "front line", dealing with children and adolescents, and interacting with their families and communities, are well positioned to recognize, investigate and help prevent environmentally related diseases and as such in a strategic position to collect data, undertake research, stimulate decision-makers to take action, and promote the education of family members and the general public. However, these groups are currently not adequately trained in this area.

The WHO department for Public Health and Environment provides excellent resources for assisting in the training of health care professionals in the front line, especially those in developing countries, who aim to increase their knowledge and understanding of children and environmental health. In addition, some tertiary institutions are now providing on-line education in Children's Environmental Health. A more adequately trained work force is required to reduce the environmental burden of disease suffered by children.

## Competing interests

The authors declare that they have no competing interests.

## Authors' contributions

TG compiled information and ideas and wrote the final commentary. JP provided background material. PS contributed pediatric medical knowledge and expertise and edited the manuscript.. All authors read and approved the final manuscript.

## About the authors

Tania G Gavidia

Tania obtained a Masters in International Health in 2008 and works as a research assistant at the WHO Collaborating Centre for research on children's environmental health in Perth, Australia. She has previously worked in developing countries assessing causes of disease of vulnerable populations.

Peter D Sly

Professor Peter Sly is the Director of a WHO Collaborating Centre researching the environmental causes of childhood illnesses. He also heads the Division of Clinical Sciences at the Telethon Institute for Child Health Research, Perth, Australia. As a paediatric respiratory physician, Professor Sly has extensive experience on the clinical management of childhood asthma, allergies and other diseases linked to the environment. As a researcher, he works on respiratory physiology and has forged an international reputation in the areas of infant lung function testing, the measurement of lung tissue mechanics, and on markers of respiratory disease in small children.

Jenny Pronczuk de Garbino

Jenny Pronczuk de Garbino is a Medical Officer leading the technical activities on children's environmental health in the Department of Public Health and Environment. She trained as a Physician and was appointed Head Professor of Clinical Toxicology and Director of the National Poisons Center before joining WHO in 1991 to work on chemical safety, global toxic outbreak alert and response, until dealing with the environmental health of vulnerable population groups, especially children.

## Pre-publication history

The pre-publication history for this paper can be accessed here:


